# Phase-Based Gait Prediction after Botulinum Toxin Treatment Using Deep Learning

**DOI:** 10.3390/s24165343

**Published:** 2024-08-18

**Authors:** Adil Khan, Omar Galarraga, Sonia Garcia-Salicetti, Vincent Vigneron

**Affiliations:** 1 Informatique, Bio-Informatique et Systèmes Complexes (IBISC) EA 4526, Univ Evry, Université Paris-Saclay, 91020 Evry, France; vincent.vigneron@univ-evry.fr; 2 Department of Computer Science, Sukkur IBA University, Sukkur 65200, Sindh, Pakistan; 3 UGECAM Ile-de-France, Movement Analysis Laboratory, 77170 Coubert, France; omar.galarraga@ugecam.assurance-maladie.fr; 4 SAMOVAR, Télécom SudParis, Institut Polytechnique de Paris, 91120 Palaiseau, France

**Keywords:** botulinum toxin, clinical gait analysis, deep learning, gait rehabilitation, long short-term memory, multi-task learning

## Abstract

Gait disorders in neurological diseases are frequently associated with spasticity. Intramuscular injection of Botulinum Toxin Type A (BTX-A) can be used to treat spasticity. Providing optimal treatment with the highest possible benefit–risk ratio is a crucial consideration. This paper presents a novel approach for predicting knee and ankle kinematics after BTX-A treatment based on pre-treatment kinematics and treatment information. The proposed method is based on a Bidirectional Long Short-Term Memory (Bi-LSTM) deep learning architecture. Our study’s objective is to investigate this approach’s effectiveness in accurately predicting the kinematics of each phase of the gait cycle separately after BTX-A treatment. Two deep learning models are designed to incorporate categorical medical treatment data corresponding to the injected muscles: (1) within the hidden layers of the Bi-LSTM network, (2) through a gating mechanism. Since several muscles can be injected during the same session, the proposed architectures aim to model the interactions between the different treatment combinations. In this study, we conduct a comparative analysis of our prediction results with the current state of the art. The best results are obtained with the incorporation of the gating mechanism. The average prediction root mean squared error is 2.99° ($R^{2}$ = 0.85) and 2.21° ($R^{2}$ = 0.84) for the knee and the ankle kinematics, respectively. Our findings indicate that our approach outperforms the existing methods, yielding a significantly improved prediction accuracy.

## 1. Introduction

Musculoskeletal and neurological diseases lead to diminished quality of life [1]. These diseases may be associated with spasticity, a motor disorder that causes excessive tendon jerks due to hyper-excitability of the stretch reflexes [2]. Medical doctors recommend rehabilitation for such deficiencies, in addition to pharmacologic treatment. In particular, spasticity is usually treated with intramuscular Botulinum Toxin Type A (BTX-A) injections that enhance lower and upper limb function [3,4]. It is essential to ensure that the total dose of BTX-A administered to the patient and the doses on each treated muscle do not exceed the recommended maximal doses [5]. BTX-A injections should be separated at least three months apart due to their dangers and reversible effects on muscular function. A good BTX-A outcome can enhance and accelerate the rehabilitation process, but a bad outcome could slow down and/or limit the patient’s recovery. Therefore, optimizing BTX-A treatment by choosing the correct muscles to be treated and the dose distribution is a complex and crucial task that requires careful patient assessment. Anticipating the most likely outcome of a specific BTX-A treatment could help clinicians find the most adapted treatment combination (muscle and doses) for each patient. This could also potentially enhance the patient’s participation in the treatment and rehabilitation process.

Clinical Gait Analysis (CGA) is considered for treatment decisions, along with a medical history and physical examination. Based on a biomechanical interpretation of instrumental measures, CGA examines walking issues and suggests causes [6]. CGA data are clinically reliable if quality standards are met [7]. Scientific research has shown that CGA helps assess and treat neurological diseases such as Cerebral Palsy (CP) [8], post-stroke hemiparesis [4] and Multiple Sclerosis (MS) [9].

Deep Neural Networks (DNN) have excelled in clinical decision-making [10,11]. Deep Learning (DL) has been used in several CGA works to predict gait trajectories, primarily for healthy gaits [12,13,14,15]. Most works on pathological gaits tackle classification [16,17,18,19]. Among them, a few studies predict the gait trajectory some timestamps ahead [20,21], but they do not attempt to predict the post-treatment gait trajectory. Kolaghassi et al. [22] studied the abnormal walking patterns of children with neurological disorders. They used Long Short-Term Memory (LSTM) and a Convolutional Neural Network (CNN) to predict future hip, knee, and ankle trajectories up to 200 ms. Su et al. [20] used LSTM to predict gait trajectories and the five gait phases (loading response, mid-stance, terminal stance, pre-swing, and swing) to help design exoskeletons. Karakish et al. [12] implemented more reliable and simple Artificial Neural Networks (ANNs) to predict the gait trajectories using shank and foot IMU data. Four subjects were used for training, and a fifth was used for testing (200 ms future trajectories). Ding et al. [13] proposed a model for motion prediction of a subject using the motion of complementary limbs. They implemented LSTM with an attention mechanism.

The goal of this research is to enhance the prediction of the post-treatment (with BTX-A) gait signals using DL models to help clinicians with therapeutic decision-making. Our contribution aims to predict the gait trajectory after BTX-A treatment and determine how different treatments might be combined. It is based on an architecture that can handle sparse inputs (treatment data) and construct a more robust model by sharing information between sub-models representing different treatments [21]. In our past study [23], we compared serial and parallel architectures. The best one was made up of parallel Bi-LSTM-shaped sub-models. Each sub-model was paired with a treatment (injected muscle). Those models learn to map gait sequences well before and after treatment [23].

In this work, we split the learning process into stance and swing phases for the parallel architectures in [23]. Indeed, as we work on pathological gaits, the signals show more variance than normal gaits [24]. We note that the duration of the stance phase is often longer for patients than for healthy subjects; for example, in post-stroke hemiparesis [25]. The stance and swing phases obey different biomechanical constraints; therefore, we train separate models on each phase to enhance post-treatment CGA prediction. This strategy increases the global model’s ability to accurately predict the complete post-treatment gait cycle. We show significant improvements in the prediction quality of pathological gait cycles [23].

## 2. Materials and Methods

### 2.1. Dataset Description

This study gathered data at the Movement Analysis Laboratory at the Rehabilitation Center of UGECAM Coubert (France) using a four-camera CX1 optoelectronic Codamotion system operating at a frequency of 100 Hz. The participants were adults with varying gait abnormalities: MS, stroke, CP, Spinal Cord Injury (SCI), and Traumatic Brain Injury (TBI). The $N_{pat}$ = 43 patients (26 males and 17 females) received BTX-A injections for spasticity treatment. In this retrospective analysis, the patients participated in clinical activities. The institution’s research ethics committee approved the use of these data. The patients were informed about the research and did not object to the use of their data. The patients had undergone treatment via injections into their lower limbs, with $N_{uni}$ = 19 patients (44.18%) affected unilaterally (10 right limbs and 9 left limbs) and $N_{bil}$ = 24 patients (55.82%) affected bilaterally. Their ages ranged from 21 to 75 years old. The time lag between pre- and post-treatment CGA was between 3 and 6 weeks. A total of nineteen muscles were injected, but we selected four frequently injected muscles: soleus, gastrocnemius (medial and lateral), semitendinosus, and rectus femoris. A fifth category, “other muscles”, grouped all the other treated muscles (see Table 1). There were 28 combinations of BTX-A injections into these four muscles. A treatment binary code vector was attributed to each lower limb:

$$s^{j}={\left(s_{1}^{j},\ldots ,s_{c}^{j}\right)}^{T},s_{i}^{j}\in \left\{0,1\right\},i=1\ldots c\;\left(c=5\;\mathrm{as}\;\mathrm{shown}\;\mathrm{in}\;\mathrm{Table}\;1\right)$$

where $s_{i}^{j}=1$ if muscle *i* was injected in limb *j*, 0 otherwise, and $d^{j}={\left(d_{1}^{j},\ldots ,d_{5}^{j}\right)}^{T},d_{i}^{j}\in \left\{0,1\right\}$ is a binary vector for the disease of the patient’s limb *j*. This study included five diseases: CP, MS, TBI, SCI, and stroke. ^*T*^ is the transpose operator.

### 2.2. Dataset Preparation

**Figure 1:**
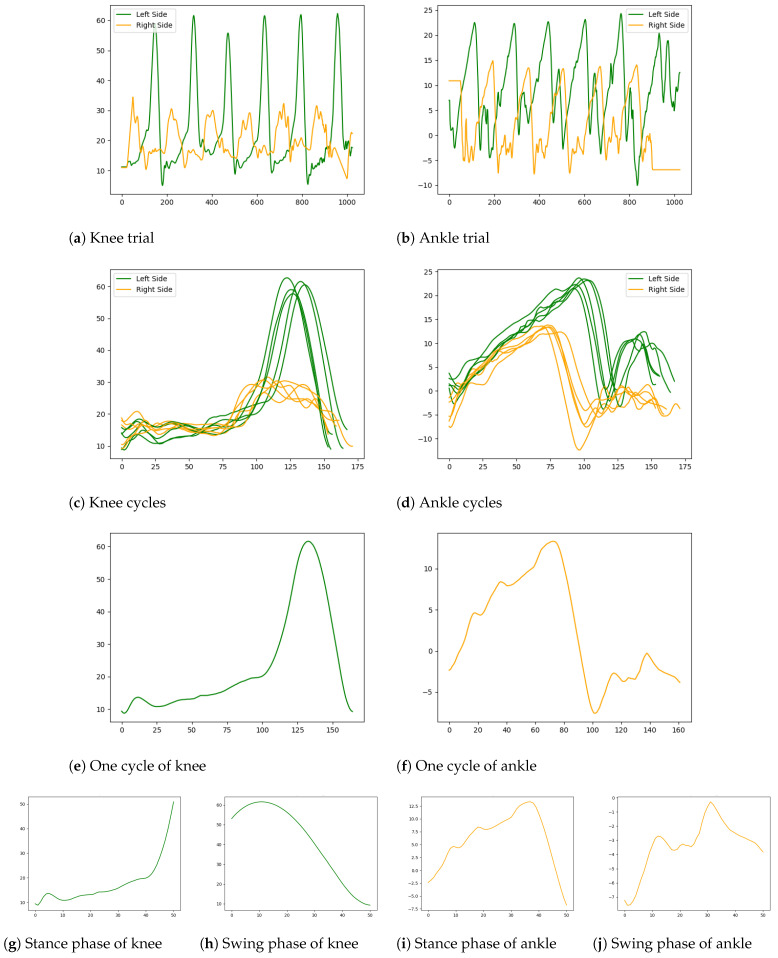
Process of converting each patient’s trial into normalized gait phases (stance and swing). (**a**,**b**) knee and ankle trials of a random Patient i, respectively; (**c**,**d**) knee and ankle cycles of Patient i extracted from their trials; (**e**,**f**) One cycle of the knee and ankle joints, respectively; (**g**,**i**) normalized stance phase of knee and ankle joints from given cycles, respectively; (**h**,**j**) normalized swing phase of knee and ankle joints, respectively.

**Figure 2:**
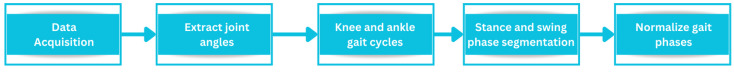
Flowchart of data preprocessing. We acquired the trajectories of patient trials from the database and extracted all cycles from the trials for the knee and ankle joints. After that, we selected each cycle and segmented them into stance and swing phases. Finally, we normalized the phases.

The participants were recorded walking in a straight line, with or without technical aids (i.e., cane, rollator, tripod, etc.), through a 10 m-long laboratory room. The patients wore anatomical markers, whose coordinates were tracked in 3D using four sensors. The patients walked back and forth throughout the gait hallway (trials). Depending on the patient’s capability, multiple trials of each patient were recorded at 100 Hz. The 3D gait kinematics were computed following the recommendations of the International Society of Biomechanics [26] based on the marker data. Each trial was divided into cycles and then segmented into the stance phase, from initial contact to toe-off, and the swing phase, from toe-off to subsequent initial contact (see Figure 1). Gait events (initial contacts and toe-offs) were detected from force platform data and automatically extracted by the HPA algorithm [27]. A human expert validated and modified all the gait events (when needed). The process of extracting cycles from trials to normalized phases is shown in Figure 2.

We considered a person’s right and left cycles as different samples. Each pre-treatment cycle phase was associated with a target post-treatment cycle’s corresponding phase of the same patient, leading to n = 2518 samples in total. Note that the number of cycles per patient varied from one patient to another.

Patient data were recorded for the following five joints in 3D: pelvis, hip, knee, ankle, and foot. This study only considered the knee and ankle kinematics in the sagittal plane.

The kinematic data of each phase were resampled and normalized to 51 points following standard procedures in CGA [28]. Therefore, DL models could be trained on sequences of the same length. For any patient’s limb *j*, the input vector is an angular time series $x^{j}={\left(x_{1}^{j},\ldots ,x_{m}^{j}\right)}^{T}\in {\left[-180,+180\right]}^{m}$, and the target vector is $y^{j}={\left(y_{1}^{j},\ldots ,y_{m}^{j}\right)}^{T}$, with m=51×2=102. Let $D={\left\{x^{j},y^{j},d^{j},s^{j}\right\}}_{j=1}^{n}$ be the input–target training set.

The data were centered and reduced by the standard deviation for normalization purposes. The goal was to use g(x) to make a model that maps $\hat{Y}=g\left(x\right)$, where $\hat{Y}$ is an estimation of y.

### 2.3. Description of Models

In our previous study [23], seven models were developed for prediction. Four were Bi-LSTM-based parallel models, and the others were serial models. The parallel models achieved better prediction results in 38 patients. LSTM [29] is good at predicting time series and can retain information for a long period of time. It has a hidden state $h_{t}$ and a cell state $c_{t}$ of the same size as the input series $x_{t}$. The cell state $c_{t}$ is the model’s memory. The hidden state $h_{t}$ is the model’s prediction of $x_{t}$.

We use the best models from our previous study [23], which are based on a Bi-LSTM. Bi-LSTM combines two LSTM models trained simultaneously: one on the forward input series and the other on the reverse input series, starting with the last input and moving on to the next-to-last, and so forth.

**Figure 3:**
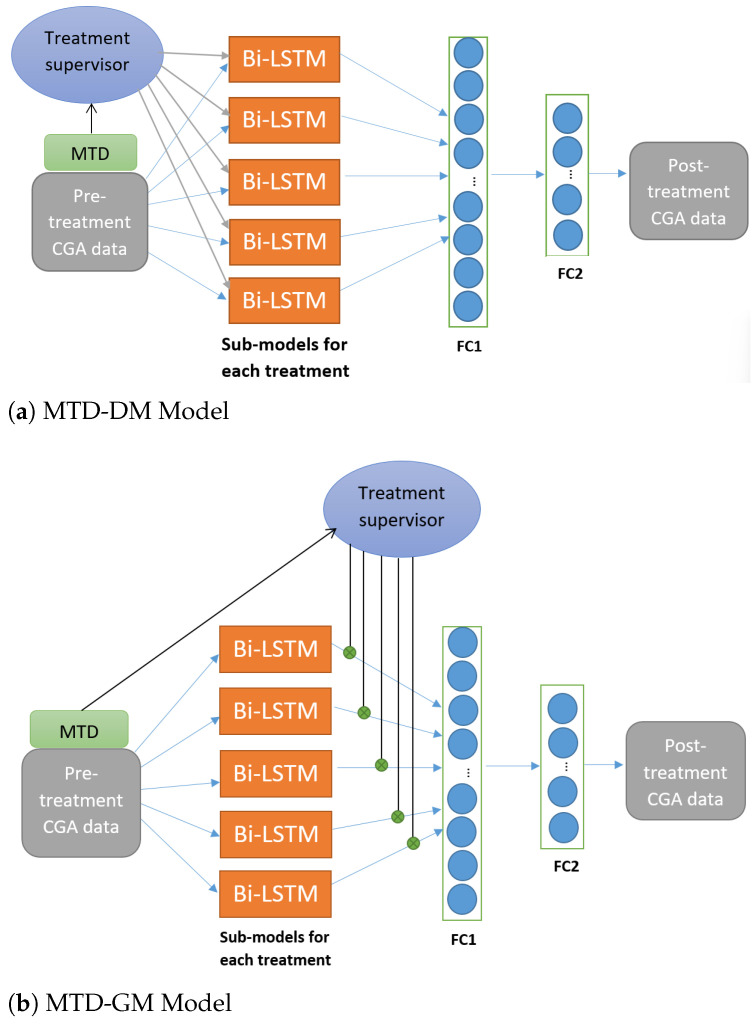
(**a**) Multi-task learning architecture with Bi-LSTM sub-models: processing of MTD in each sub-model. (**b**) Multi-task learning architecture with Bi-LSTM sub-models: incorporating MTD through a gating mechanism.

In both models (see Figure 3), we use five parallel layers of Bi-LSTM. Each layer is responsible for a treatment, as reported in Table 1. The five treatments correspond to the five categories of injected muscles. Each Bi-LSTM layer had 51 units. Note that each unit receives a pair of inputs for the knee and ankle, respectively.

We train separate models for the stance and swing phases. For comparison with our previous work [23], we combine both predictions using the proportion of stance and swing phases of the pre-treatment gait cycle. We retrieve the prediction for a complete cycle of the knee and ankle joints.

**MTD-driven model (MTD-DM):** The input vectors x and s are sent to the five Bi-LSTM sub-models in this architecture. The pre-treatment knee and ankle kinematic signals are represented by vector x. The Medical Treatment Data (MTD) are represented by vector s. We initialize the cell states of the LSTM as 0. The MTD (vector s) is handled by the treatment supervisor and was used to set the values of the hidden states *h*. For example, if a patient had injections in muscles 1 and 3 (Table 1), then the states $h_{1,t}$, $h_{2,t}$ in Bi-LSTM, layers 1 and 3 are set to 1, and the other layers’ (2, 4, and 5) hidden states are set to 0. The results of the five Bi-LSTM sub-models are concatenated to form a single tensor ‘output’, a one-dimensional vector, and reshaped. The reshaped output is then processed through two subsequent fully connected layers (FC1 and FC2) to predict the post-treatment kinematics (see Figure 3a).

**MTD-gated model (MTD-GM):** This architecture uses a gating mechanism to handle MTD. Instead of passing the MTD as a hidden state of each sub-model, the treatment supervisor is exploited downstream, multiplying each sub-model’s output by the associated binary value of the MTD. Figure 3b shows that if there is a treatment, it will be used in the model, but if there is none, it will be neglected (multiplied by 0). The results of the remaining Bi-LSTM sub-models are concatenated and reshaped. The reshaped output is then processed through two subsequent fully connected layers (FC1 and FC2) to predict the post-treatment kinematics (see Figure 3b). Leave-one-out cross-validation was used to assess model performance. For each iteration, $N_{train}$ = $N_{pat}-1$ patients were used to train the model, and one was used to test it. During the training process, mini-batches with 16 samples were used. DL models were trained based on the mean square error (MSE) loss function, controlled by the Adam optimizer [30]. RMSE [31], Standard Error (SE) [32], and the coefficient of determination ($R^{2}$) [33] were used to evaluate the performance of the proposed models.

## 3. Results

A total of 43 participants were included in this study, whereas 38 were included in our previous work [23], and five new ones were added. We evaluated the two MTL models on the new dataset using the above-mentioned evaluation metrics.

### 3.1. Analysis of Stance Phase and Swing Phase

Table 2, Table 3 and Table 4 report the performance of both models in predicting the post-treatment gait kinematics of the stance phase, swing phase, and complete gait cycle concerning the disease. The bold entries in the following tables represent the best predictions (lowest RMSE and highest $R^{2}$ scores). We notice that the performances of both models are equivalent in the stance phase for the knee and ankle joints (average RMSE between 1.79° and 3.17°). On the contrary, for the swing phase, MTD-GM is much better than MTD-DM, decreasing the RMSE from 3.08° and 7.01° (knee) for MTD-DM to 2.43° and 3.89° (ankle) for MTD-GM. With the MTD-GM, in the swing phase, the $R^{2}$ score is better for the knee than for the ankle. It is the opposite of the ankle for the stance phase.

### 3.2. Analysis of Complete Cycle

As reported in Table 4, we combine the stance and swing phase predictions to obtain the prediction for each complete cycle. We note that the MTD-GM outperforms MTD-DM, showing much better $R^{2}$ scores for the knee (higher than 0.8) and ankle (higher than 0.7). Table 5 reports the overall prediction results on both joints for MTD-DM and MTD-GM. Our results improve the $R^{2}$ scores, which become higher than 0.9 in all cases. We also note that the $R^{2}$ score is increased for all groups of diseases, because combining both series changes the variance of the whole series.

**Figure A1:**
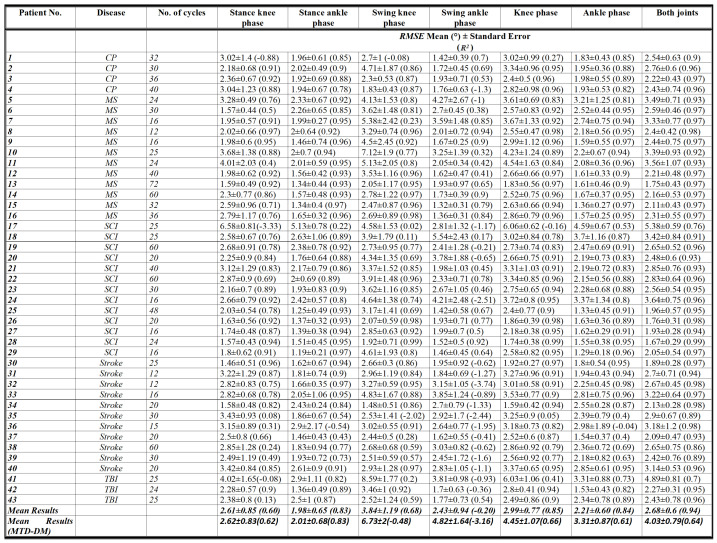
Detailed results of all patients using the MTD-GM model.

Finally, the detailed results of MTD-GM for each patient per phase and joint and on both joints together are available in the Appendix A (show Figure A1). An average RMSE predicted by MTD-GM (all patients) for the stance phase of the knee, stance phase of the ankle, swing phase of the knee, swing phase of the ankle, complete knee, and complete ankle are 2.61°, 1.98°, 3.84°, 2.43°, 2.99°, and 2.21°, respectively.

**Table 2:** Performance of both models in predicting the post-treatment gait trajectories of the stance phase with respect to different diseases. Bold entries denote the best predictions: lowest average RMSE and maximum $R^{2}$ over all limbs for a given disease.

Model	Model Type	Body Joint	Spinal Cord Injury (SCI)	Multiple Sclerosis (MS)	Stroke	Cerebral Palsy (CP)	Traumatic Brain Injury (TBI)
			**No. of Patients**				
			**13**	**12**	**11**	**4**	**3**
			**No. of Cycles**				
			**400**	**351**	**260**	**138**	**74**
			**RMSE Mean (°) ± Standard Error**				
			**$R^{2}$ Score**				
**MTD-DM**	**MTL, 5 Bi-LSTMs**	Knee	2.6 ± 0.76	2.48 ± 0.73	**2.65 ± 0.78**	**2.62 ± 1.08**	3.17 ± 1.23
			0.54	0.79	**0.64**	**0.62**	0.18
		Ankle	2.09 ± 0.79	1.83 ± 0.64	2.02 ± 0.5	1.99 ± 0.8	2.44 ± 0.78
			0.87	0.93	0.67	0.85	0.84
**MTD-GM**	**MTL, 5 gated Bi-LSTMs**	Knee	**2.59 ± 0.84**	**2.48 ± 0.7**	2.7 ± 0.78	2.65 ± 1.08	**2.89 ± 1.03**
			**0.52**	**0.80**	0.62	0.46	**0.32**
		Ankle	**2.09 ± 0.75**	**1.79 ± 0.61**	**2.01 ± 0.49**	**1.96 ± 0.82**	**2.25 ± 0.75**
			**0.85**	**0.93**	**0.68**	**0.85**	**0.86**

**Table 3:** Performance of both models in predicting post-treatment gait trajectories of the swing phase with respect to different diseases. Bold entries denote the best predictions: lowest average RMSE and maximum $R^{2}$ over all limbs for a given disease.

Model	Model Type	Body Joint	Spinal Cord Injury (SCI)	Multiple Sclerosis (MS)	Stroke	Cerebral Palsy (CP)	Traumatic Brain Injury (TBI)
			**No. of Patients**				
			**13**	**12**	**11**	**4**	**3**
			**No. of Cycles**				
			**400**	**351**	**260**	**138**	**74**
			**RMSE Mean (°) ± Standard Error**				
			**$R^{2}$ Score**				
**MTD-DM**	**MTL, 5 Bi-LSTMs**	Knee	6.23 ± 2.05	7.01 ± 2.2	7.0 ± 1.73	6.72 ± 2.29	6.8 ± 1.17
			0.31	0.59	−1.56	−3.86	0.26
		Ankle	5.15 ± 2.05	5.15 ± 2.2	4.36 ± 1.73	5.3 ± 2.29	3.08 ± 1.17
			−3.35	−1.18	−5.16	−4.36	−1.34
**MTD-GM**	**MTL, 5 gated Bi-LSTMs**	Knee	**3.52 ± 1.2**	**3.89 ± 1.24**	**2.85 ± 1.04**	**2.88 ± 1.49**	**4.86 ± 1.28**
			**0.72**	**0.84**	**0.52**	**0.63**	**0.57**
		Ankle	**2.62 ± 1.2**	**2.29 ± 1.24**	**2.63 ± 1.04**	**1.71 ± 1.49**	**2.43 ± 1.28**
			**0.06**	**0.53**	**−1.45**	**0.15**	**−0.25**

### 3.3. Visualizing Predictions of MTD-GM

**Figure 4:**
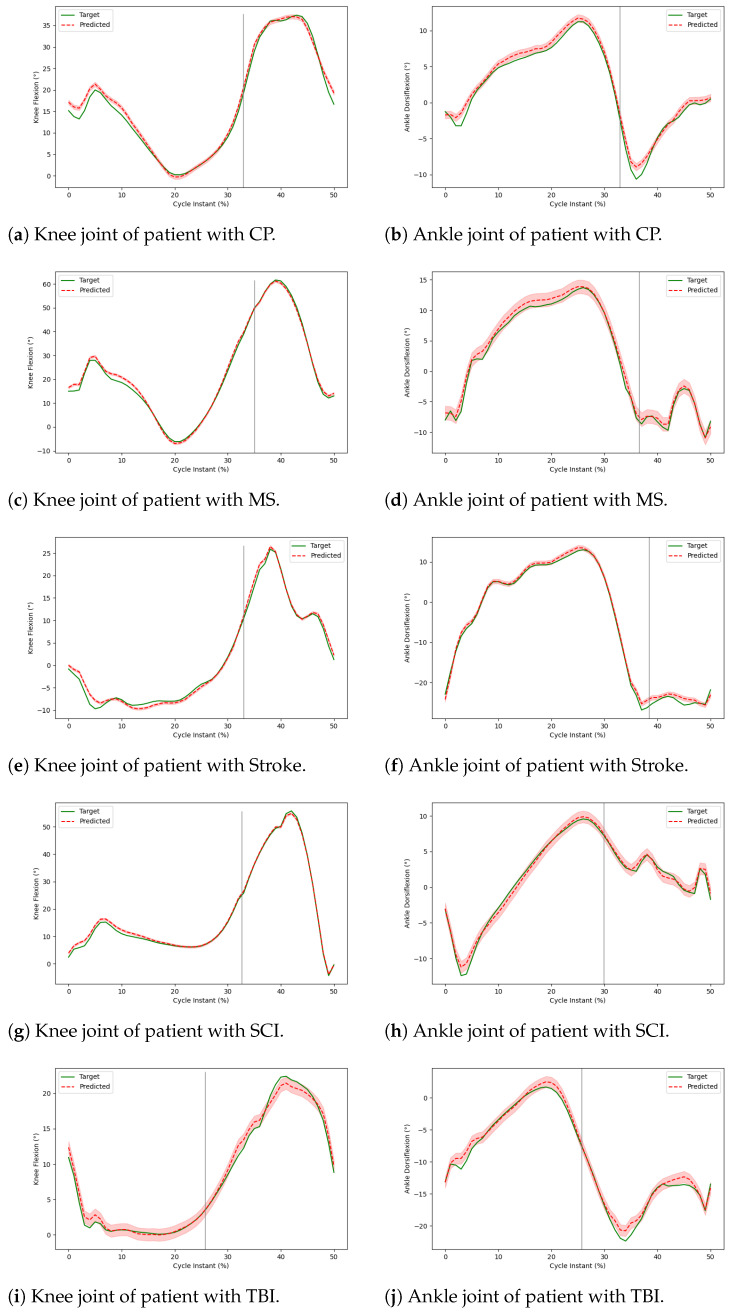
Comparison of MTD-GM prediction and post-treatment kinematics for a patient with a given disease.

Figure 4 represents the average predictions by the MTD-GM model across all cycles of five patients, each suffering from a different disease. The ankle dorsiflexion or knee flexion is shown on the Y-axis, and the patient’s gait cycle is shown on the X-axis. Figure 4a,b represent the average predictions of the CP patient, Figure 4c,d represent the average predictions of the MS patient, Figure 4e,f represent the average predictions of the stoke patient, Figure 4g,h represent the average predictions of the SCI patient, and Figure 4i,j represent the average predictions of the TBI patient. In all these graphs, we observe that each patient’s average predicted gait trajectory is very close to the average actual trajectory. Hence, it shows that our model performs well in predicting the post-treatment gait trajectory of patients with disabilities, as reported in Table 2, Table 3 and Table 4.

**Table 4:** Performance of both models in predicting the post-treatment gait trajectories of a complete cycle with respect to different diseases. Bold entries denote the best predictions: lowest average RMSE and maximum $R^{2}$ over all limbs for a given disease.

Model	Model Type	Body Joint	Spinal Cord Injury (SCI)	Multiple Sclerosis (MS)	Stroke	Cerebral Palsy (CP)	Traumatic Brain Injury (TBI)
			**No. of Patients**				
			**13**	**12**	**11**	**4**	**3**
			**No. of Cycles**				
			**400**	**351**	**260**	**138**	**74**
			**RMSE Mean (°) ± Standard Error**				
			**$R^{2}$ Score**				
**MTD-DM**	**MTL, 5 Bi-LSTMs**	Knee	4.18 ± 0.97	4.58 ± 1.11	4.58 ± 1.09	4.82 ± 1.23	4.26 ± 0.77
			0.70	0.85	0.69	−0.17	0.72
		Ankle	3.61 ± 0.99	3.35 ± 0.8	2.92 ± 0.85	3.6 ± 1.02	2.86 ± 0.73
			0.55	0.81	0.47	0.44	0.75
**MTD-GM**	**MTL, 5 gated Bi-LSTMs**	Knee	**3.05 ± 0.7**	**3.08 ± 0.69**	**2.9 ± 0.79**	**2.88 ± 0.99**	**3.28 ± 0.78**
			**0.80**	**0.93**	**0.88**	**0.80**	**0.80**
		Ankle	**2.37 ± 0.8**	**2.02 ± 0.54**	**2.3 ± 0.44**	**1.92 ± 0.72**	**2.44 ± 0.49**
			**0.83**	**0.94**	**0.70**	**0.86**	**0.82**

**Table 5:** Performance of both models for both joints in predicting the post-treatment gait trajectories with respect to different diseases. Bold entries denote the best predictions: lowest average RMSE and maximum $R^{2}$ over all limbs for a given disease.

Model	Model Type	Body Joint	Spinal Cord Injury (SCI)	Multiple Sclerosis (MS)	Stroke	Cerebral Palsy (CP)	Traumatic Brain Injury (TBI)
			**No. of Patients**				
			**13**	**12**	**11**	**4**	**3**
			**No. of Cycles**				
			**400**	**351**	**260**	**138**	**74**
			**RMSE Mean (°) ± Standard Error**				
			**$R^{2}$ Score**				
**MTD-DM**	**MTL, 5 Bi-LSTMs**	**Knee and Ankle**	4.02 ± 0.76	4.12 ± 0.79	3.91 ± 0.79	4.33 ± 1.02	3.76 ± 0.61
			0.87	0.91	0.89	0.77	0.86
**MTD-GM**	**MTL, 5 gated Bi-LSTMs**		**2.78 ± 0.61**	**2.65 ± 0.53**	**2.65 ± 0.57**	**2.48 ± 0.75**	**2.97 ± 0.57**
			**0.94**	**0.96**	**0.95**	**0.94**	**0.90**

**Figure 5:**
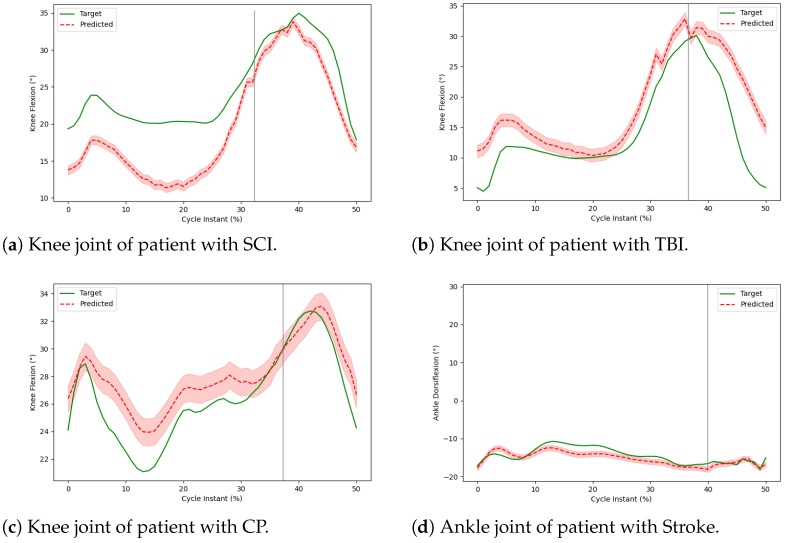
Outliers.

However, the CGA kinematics were difficult to predict for a few patients’ post-treatment. In Figure 5a–d, we report that the post-treatment cycles for these patients show more variability between cycles than before treatment. This is not the case for most of the other patients. We investigate this phenomenon below.

Figure 5a shows the trajectories of the right limb of a patient with SCI. The knee kinematics of this patient show more inter-cycle variability than most patients. The MTD-GM model predicted this patient’s post-treatment kinematics with an average RMSE of 6.06° and an $R^{2}$ value of −0.16 for the knee joint. This is worse than the average RMSE of 3.05°. We also notice that the stance phase’s prediction error is higher than the swing phase. Figure 5b shows the trajectories of a TBI patient with an issue in his left limb. The post-treatment gait kinematics of the knee joint of this patient were more intra-variant. The prediction for this patient was also not good, with an RMSE of 6.03° and an $R^{2}$ = 0.41 for the knee joint. Figure 5c shows the trajectories of a patient with CP. Both the pre- and post-treatment gait kinematics of this patient showed high variance between cycles. The prediction of this patient shows an average RMSE of 3.02° and an $R^{2}$ score of 0.27 for the knee joint. It is difficult for our DL models to predict the post-treatment gait trajectories, which show significant variance. Figure 5d shows the trajectories of a stroke patient with an issue in his lower limbs. His kinematics were also more inter- and intra-variant, with a prediction of an average RMSE of 2.98° and $R^{2}$ = −0.04 for the knee joint.

## 4. Comparison between Cycle-Based Prediction, Phased-Based Prediction, and Related Works

It is difficult to compare our results to other research, because this is the first time that the entire knee and ankle kinematic signals in the sagittal plane have been predicted for BTX-A treatment, except for in ref. [23]. In this study, we worked on the kinematic prediction per phase (stance and swing) of each gait cycle, while in [23], the complete cycles were considered. The results of the total cycles of this study and the previous study are compared in Table 6. It is important to note that five additional patients are considered in this work; thus, the dataset is similar but nonidentical. For MS and TBI diseases, we recruited the same patients in both studies, but the difference in the prediction performance is evident. We can see that the $R^{2}$ scores for stroke, TBI, and CP were negative in [23]. For our new approach, there was an improvement, achieving more than 0.7 in the $R^{2}$ score. Furthermore, we compare our results with those described by [4] for forecasts of peak knee and ankle flexion in the sagittal plane for rectus femoris BTX-A injection in stroke patients with see [Table 6]. In this case, our proposed method (MTD-GM) for stroke had a higher $R^{2}$ score for knee flexion and ankle dorsiflexion. However, since the models were not trained and tested using the same databases, this comparison should be interpreted with care. In addition, we also compare how well our model predicted the post-treatment kinematics for patients with CP. Even though the proposed methods were not tested on the same databases, their results were better than the postoperative predictions for CP made by Galarraga et al. [34], Niiler et al. [35], and Niiler [36], as shown in Table 6. MTD-GM predicted knee flexion and ankle dorsiflexion with an average RMSE of 2.9° and 1.92°, respectively.

## 5. Discussion and Conclusions

In this study, we used two DL models using Bi-LSTM modules to predict the effects of BTX-A injections on gait kinematics. As far as we know, except for our previous work [23], no other study in the literature has used DL to look at this specific prediction task. The prediction task we assess in this work is more challenging than predicting normal gait due to the high variability between and within subjects in pathological gait.

DL architectures allow information about medical treatments to be added to the model. Instead of performing a simple pre-to-post regression task, previous results show that adding information about the treatment (i.e., which muscles were treated using BTX-A) improves performance [23].

In this work, we predict knee and ankle kinematics in the sagittal plane, but we perform this prediction per gait phase separately and only by exploiting the best DL architectures proposed in [23]. These best architectures are based on Bi-LSTM sub-models. The best DL model incorporates the presence or absence of treatments based on a gating mechanism (MTD-GM model). This model predicts post-treatment gait kinematics with a lower RMSE and higher $R^{2}$ score than the MTD-DM model and for all the considered pathologies. MTD-DM introduces treatments by initializing the hidden units of each Bi-LSTM sub-model. Instead, we randomly initialize hidden units in MTD-GM and apply the gating mechanism on the outputs of the Bi-LSTM sub-models, each devoted to a unique muscle treatment.

In our previous work [23], this model (gated model) was not the best in all situations. This improvement is due to the approach proposed in this work: splitting the learning process of each DL architecture between the stance and swing phases. This proposal allows the model to cope with the high variance present in pre-treatment and post-treatment gait between individuals and within each individual. We notice the effectiveness of our approach by exploiting the advantages of DL architectures on each gait phase separately. We note that the gated model has a relative improvement of around 55% on both joints. We have thus proven the increased capability of the global model (after combining the predictions of both phases) in accurately predicting the complete gait cycles after treatment.

If we examine the results per phase, for the stance phase, both models’ performances (MTD-DM and MTD-GM) are equivalent. For the swing phase, the difference between both models is essential. The gated model (MTD-GM) is much better: it often divides the RMSE by a factor of 2. The $R^{2}$ score is generally good for the knee (higher than 0.7) but close to 0 for the ankle. Moreover, no proposed model adequately explained the variance of the swing phase of the ankle joint (note that MTD-DM performed badly, as shown by negative $R^{2}$ scores).

We conclude that using treatment data to initialize hidden variables in each Bi-LSTM sub-model is not enough to cope with the high variance encountered in the data, especially in the swing phase. Indeed, for this phase, we remark that a more drastic treatment supervisor for the outputs of the Bi-LSTM sub-models is required. In this work, we do not address the segmentation between phases of gait cycles; instead, we focus on the prediction quality of our models by exploiting the segmentation of the pre-treatment gait cycles on the post-treatment gait cycles. In future work, we will study the impact of considering treatment dosage information. We expect to refine our predictions of post-treatment kinematics by including this information in our DL architecture.

## Figures and Tables

**Table 1:** Considered injected muscles and their frequencies in the database.

Muscle Number	Muscle/Category	Injections in Patient	
		Number	Proportion
1	Soleus	53	28.3%
2	Gastrocnemius (medialis and/or lateralis)	51	27.2%
3	Rectus femoris	22	11.7%
4	Semitendinosus	14	7.4%
5	Other muscle	47	25.1%

**Table 6:** Performance comparison to related works. Bold entries denote the best predictions: lowest average RMSE and maximum $R^{2}$ over all limbs for a given disease. * Only peak flexion and not over the whole time series.

Model	Model Type	Body Joint	Spinal Cord Injury (SCI)	Multiple Sclerosis (MS)	Stroke	Cerebral Palsy (CP)	Traumatic Brain Injury (TBI)
			**RMSE Mean (°) ± Standard Error**				
			**$R^{2}$ Score**				
**Model 1 [23]**	**MTL, 5 Bi-LSTMs**	**Knee**	7.51 ± 1.67	7.23 ± 1.69	7.14 ± 1.08	6.75 ± 1.73	5.81 ± 1.33
			0.63	0.63	0.01	0.7	0.54
		**Ankle**	5.01 ± 0.99	4.38 ± 1.15	6.85 ± 1.61	6.4 ± 1.19	4.68 ± 0.82
			0.38	0.55	−3.58	−0.01	0.4
**Model 2 [23]**	**MTL, 5 gated Bi-LSTMs**	**Knee**	7.62 ± 1.89	7.23 ± 2.01	8.02 ± 1.09	7.00 ± 2.42	5.60 ± 1.4
			0.53	0.48	0.21	0.68	0.65
		**Ankle**	4.56 ± 1.05	5.39 ± 1.37	7.14 ± 1.44	3.77 ± 1.41	4.93 ± 1.0
			0.27	0.28	−3.65	−0.16	−0.5
**MTD-DM**	**MTL, 5 Bi-LSTMs**	**Knee**	4.18 ± 0.97	4.58 ± 1.11	4.58 ± 1.09	4.82 ± 1.23	4.26 ± 0.77
			0.70	0.85	0.69	−0.17	0.72
		**Ankle**	3.61 ± 0.99	3.35 ± 0.8	2.92 ± 0.85	3.6 ± 1.02	2.86 ± 0.73
			0.55	0.81	0.47	0.44	0.75
**MTD-GM**	**MTL, 5 gated Bi-LSTMs**	**Knee**	**3.05 ± 0.7**	**3.08 ± 0.69**	**2.9 ± 0.79**	**2.88 ± 0.99**	**3.28 ± 0.78**
			**0.80**	**0.93**	**0.88**	**0.80**	**0.80**
		**Ankle**	**2.37 ± 0.8**	**2.02 ± 0.54**	**2.3 ± 0.44**	**1.92 ± 0.72**	**2.44 ± 0.49**
			**0.83**	**0.94**	**0.70**	**0.86**	**0.82**
LigReg Stroke * [4] ($R^{2}$ Score)	**Linear Regression**	**Knee**	-	-	0.24	-	-
		**Ankle**	-	-	0.43	-	-
**MLinReg CP [34] (RMSE(°))**	**Multiple Linear Regression**	**Knee**	-	-	-	9.0	-
		**Ankle**	-	-	-	7.5	-
**NN99 CP [35] (RMSE(°))**	**NN**	**Knee**	-	-	-	9.7	-
		**Ankle**	-	-	-	6.7	-
**NN01 CP [36] (RMSE(°))**	**NN**	**Knee**	-	-	-	9.2	-
		**Ankle**	-	-	-	-	-

## Data Availability

Due to the nature of this research, the participants of this study did not agree for their data to be shared publicly, so the supporting data are not available.

## Abbreviations

The following abbreviations are used in this manuscript:

AI	Artificial intelligence
ML	Machine learning
DL	Deep learning
MTL	Multi-task learning
CP	Cerebral palsy
CGA	Clinical gait analysis
LSTM	Long short-term memory
CNN	Convolutional neural network
MAE	Mean absolute error
RMSE	Root mean squared error
SE	Standard error
BTX-A	Botulinum toxin type A
TBI	Traumatic brain injury
SCI	Spinal cord injury
MS	Multiple sclerosis
CMA	Clinical movement analysis
DNN	Deep neural network
RNN	Recurrent neural network
IMU	Inertial measurement unit
HPA	High-pass algorithm
Bi-LSTM	Bi-directional LSTM
MTD	Medical treatment data

## Appendix A

The detailed results of all the patients using the MTD-GM model are listed in the following figure.
